# ‘I Can See Letters but Cannot Read Sentences’: A Case of Pure Alexia Without Agraphia Due to Left Posterior Cerebral Artery Infarction

**DOI:** 10.7759/cureus.89974

**Published:** 2025-08-13

**Authors:** Khalifa O Gnieber, Aly A Barakat, Ammar Ali Khan, Shireen Sabeeh, Syed A Kirmani

**Affiliations:** 1 Acute Medicine, Medway NHS Foundation Trust, Gillingham, GBR; 2 Internal Medicine, Medway NHS Foundation Trust, Gillingham, GBR; 3 General Internal Medicine, Medway NHS Foundation Trust, Gillingham, GBR

**Keywords:** atrial fibrillation, emergency medicine, higher cortical dysfunction, posterior cerebral artery stroke, pure alexia

## Abstract

Pure alexia without agraphia is a rare neurological condition characterised by the sudden loss of reading ability, while writing and verbal communication remain unaffected. It is typically associated with infarcts in the territory of the left posterior cerebral artery (PCA). Early recognition is essential, particularly when symptoms are subtle or atypical.

We present the case of a woman in her 60s with a history of poorly controlled hypertension, type 2 diabetes mellitus, and a previous transient ischemic attack, who presented to the same-day emergency care unit with sudden-onset difficulty reading full sentences, despite being able to recognise individual letters. She reported no other focal neurological symptoms. On examination, visual acuity, writing ability, and motor and sensory functions were preserved. A non-contrast CT brain scan showed hypoattenuation in the left occipitotemporal region, and CT angiography revealed partial thrombosis of the distal left PCA. Electrocardiography showed atrial fibrillation, suggesting a potential cardioembolic source for the infarct.

This case highlights the diagnostic challenge of posterior circulation stroke presenting as isolated reading impairment. It emphasises the importance of considering cerebrovascular causes in patients with atypical cognitive or visual disturbances. Early neuroimaging, particularly non-contrast CT brain, and evaluation for cardiac arrhythmias are crucial. Clinicians should maintain a high index of suspicion for rare stroke presentations in older patients with vascular risk factors to ensure timely diagnosis and management.

## Introduction

Reading involves a complex interaction between visual processing areas and language networks in the brain. Disruptions to this system can result in reading disorders, which are generally categorised as either developmental or acquired. Developmental dyslexia emerges in childhood and is associated with phonological deficits and altered neural activity in the temporoparietal and occipitotemporal cortices [1]. In contrast, acquired reading disorders - commonly referred to as alexia - occur following brain injury, such as stroke, trauma, or neoplasia [2].

One of the most distinctive forms of alexia is alexia without agraphia, also called pure alexia or letter-by-letter reading. It is characterised by a marked loss of reading ability while preserving writing, spelling, and spoken language skills. The dissociation between reading and writing in this condition underscores the specialised pathways in the brain responsible for visual word recognition and linguistic integration [3].

First identified by Déjerine in 1892, pure alexia is linked to lesions in the left occipitotemporal region and the splenium of the corpus callosum. This anatomical damage interrupts the transfer of visual input from the intact right visual cortex to the language-dominant left hemisphere [4]. More recent studies using advanced imaging techniques have confirmed that damage to the left fusiform gyrus - particularly an area known as the visual word form area (VWFA) - plays a critical role in the development of this syndrome [5,6].

Infarction in the left posterior cerebral artery (PCA) territory is the most common vascular cause of pure alexia, as the PCA supplies both the occipitotemporal cortex and the posterior corpus callosum. Although rare, this condition provides important insights into cortical specialisation for reading. Pure alexia without agraphia is considered uncommon, with only a limited number of well-documented cases in the literature. Several case series and reports suggest that isolated pure alexia occurs in fewer than 1% of stroke patients, though precise prevalence data are lacking [7,8].

Patients with pure alexia typically attempt to decode words by identifying each letter individually, a strategy that is slow and inefficient. They are often able to write coherently, even though they cannot read back their own writing - further highlighting the functional disconnection rather than a global language deficit [9].

Prior case reports have described pure alexia following infarcts involving the left occipitotemporal cortex and/or splenium of the corpus callosum, with varying degrees of associated deficits such as hemianopia or language impairment. However, truly isolated presentations - such as those without accompanying aphasia, agraphia, or visual field loss - remain exceptionally rare [7,8].

This report presents a case of pure alexia without agraphia following an acute ischemic infarct in the left PCA territory. The presentation was notable for an isolated inability to read, with preserved writing and language skills, emphasising the importance of considering posterior circulation stroke in patients with sudden-onset higher cognitive deficits.

## Case presentation

A 66-year-old right-handed woman presented to the same-day emergency care (SDEC) unit with an abrupt onset of reading difficulty. She reported that she could clearly recognize individual letters but was completely unable to form words or understand sentences. Notably, she retained the ability to write full, grammatically correct sentences but was unable to read what she had written moments earlier. She described this experience as disorienting, despite otherwise feeling cognitively intact.

Her past medical history included poorly controlled hypertension, type 2 diabetes mellitus, a previous transient ischemic attack, and supraventricular tachycardia. She was not on anticoagulation medication. She denied any weakness, sensory changes, speech disturbance, vertigo, visual blurring, or confusion. There were no features suggestive of aphasia or global cognitive impairment.

On initial examination, she was alert and oriented, with fluent, spontaneous speech. Cranial nerve examination was unremarkable. Visual acuity was preserved, and eye movements were full and conjugate. There were no overt visual field deficits on confrontation, though a subtle right-sided field loss could not be excluded definitively at the bedside. Motor and sensory examinations were normal, with symmetric reflexes and flexor plantar responses bilaterally. Coordination and gait were intact.

Language and cognitive assessment demonstrated preserved verbal fluency, naming, repetition, and comprehension. She was able to write dictated sentences without difficulty. However, she could not read either printed text or her own writing. Letter-by-letter decoding was possible but laborious and without comprehension. This specific dissociation between preserved writing and impaired reading, in the absence of aphasia or broader cognitive dysfunction, suggested a diagnosis of alexia without agraphia.

Laboratory workup was performed to exclude reversible metabolic causes of cognitive impairment. The patient’s laboratory tests revealed an HbA1c of 79 mmol/mol, consistent with poorly controlled diabetes. Vitamin B12, folate, full blood count, electrolytes, and renal function tests were all within normal limits.

**Figure 1:**
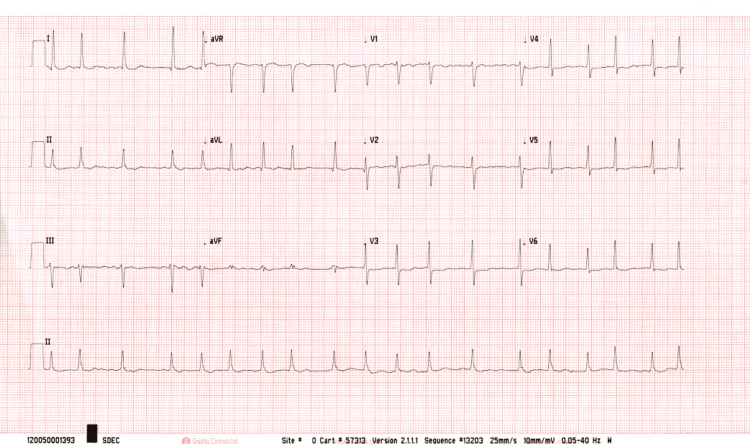
12-Lead Electrocardiogram The ECG demonstrates new-onset atrial fibrillation, characterized by irregularly irregular rhythm and absence of distinct P-waves. This arrhythmia likely served as a cardioembolic source, a recognized risk factor for posterior circulation strokes.

**Figure 2:**
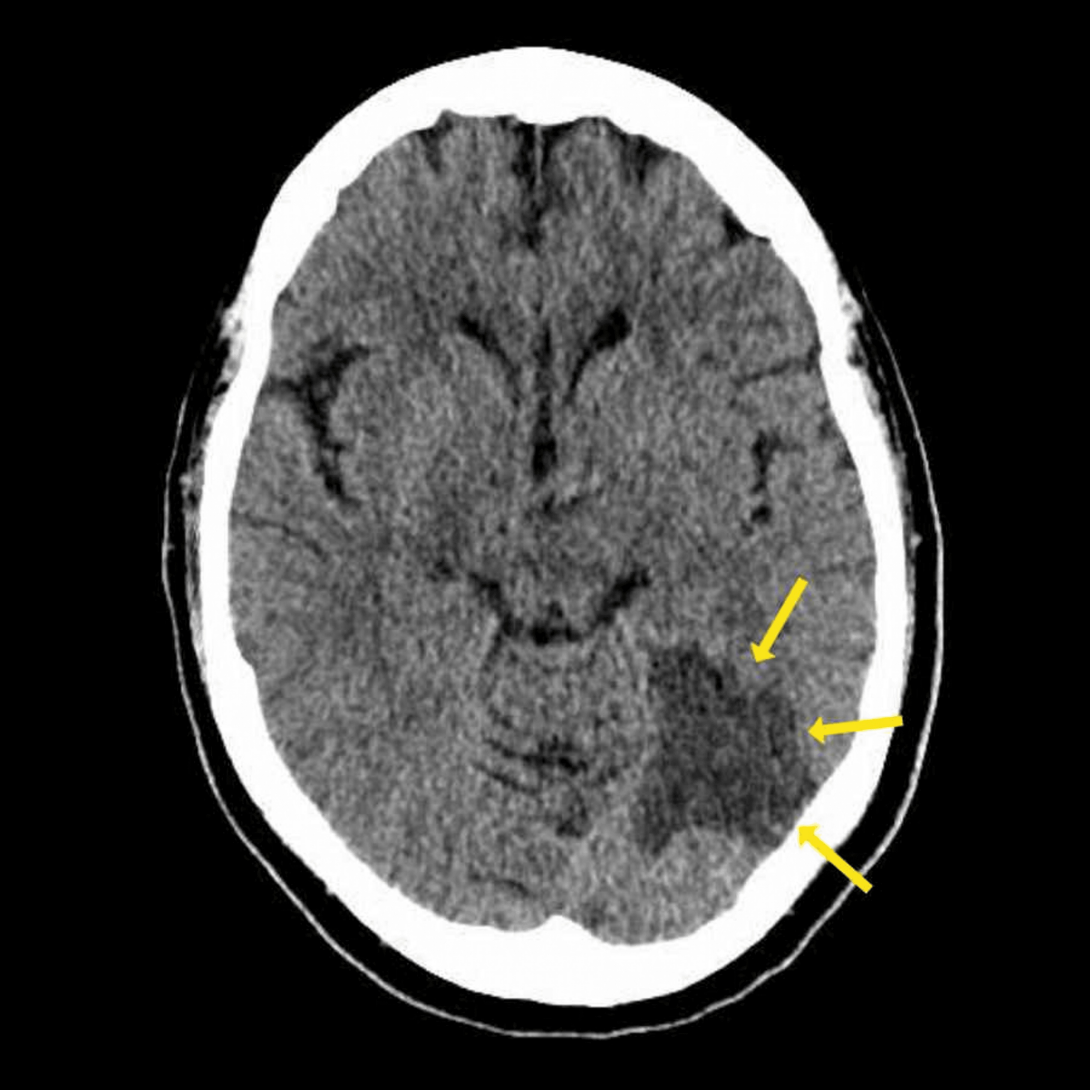
CT Head Without Contrast CT image showing loss of normal grey-white matter differentiation and hypoattenuation in the left posterior and medial temporal lobe (yellow arrows, with regional sulcal effacement, consistent with an acute or subacute infarction in left PCA territory. PCA: posterior cerebral artery

A routine electrocardiogram revealed new-onset atrial fibrillation (Figure 1). Urgent non-contrast computed tomography (CT) of the head showed an area of hypoattenuation in the left medial posterior temporal lobe, consistent with an acute infarction in PCA territory (Figure 2). CT angiography performed at the regional stroke unit showed reduced opacification of the distal segment of the left PCA, indicating partial thrombotic occlusion.

The clinical presentation and imaging findings were consistent with alexia without agraphia caused by infarction in the left PCA territory affecting the left occipitotemporal cortex and splenium of the corpus callosum. The new diagnosis of atrial fibrillation suggested a cardioembolic stroke as the underlying aetiology.

The patient was promptly admitted to the stroke unit, started on appropriate secondary prevention, and referred for community-based rehabilitation. Over time, she demonstrated gradual improvement in reading, aided by compensatory strategies such as letter-by-letter decoding.

## Discussion

Pure alexia without agraphia represents a rare and striking example of a disconnection syndrome. The hallmark clinical presentation is a complete inability to read, despite preservation of other language faculties, including spontaneous speech, comprehension, and writing. This dissociation is believed to arise from the disruption of neural pathways that connect visual inputs with language processing centers localized primarily in the dominant hemisphere [3].

In the present case, the patient’s sudden loss of reading ability - despite intact letter recognition and preserved writing skills - aligns with a lesion involving the left occipitotemporal cortex, specifically the visual word form area (VWFA). Additionally, damage to the splenium of the corpus callosum likely interrupted communication between the right visual cortex and left-hemisphere language regions. Consequently, although the right occipital lobe processed visual information normally, this input could not be transferred to language networks necessary for word recognition [4,5].

Neuroimaging findings confirmed infarction within PCA territory, corroborating the anatomical basis for the syndrome. The patient’s newly diagnosed atrial fibrillation likely contributed to a cardioembolic mechanism, a well-established cause of PCA strokes [8]. Remarkably, aside from the specific reading deficit, the neurological examination revealed no motor or sensory abnormalities. This highlights that strokes affecting higher cortical functions, especially those in the posterior circulation, may manifest with subtle or isolated cognitive impairments, underscoring the need for high clinical vigilance [9].

Epidemiological data on pure alexia without agraphia remain limited due to the condition’s rarity. Available case series estimate its occurrence in fewer than 1% of all stroke patients [7,8]. Most documented cases also include additional neurological deficits such as hemianopia or aphasia, rendering isolated pure alexia especially uncommon. This infrequency, coupled with lesion heterogeneity and variable clinical presentations, stresses the importance of comprehensive case reporting to improve recognition and understanding of this syndrome [7].

A review of the literature consistently associates pure alexia with lesions in the left occipitotemporal cortex and the splenium of the corpus callosum, typically following PCA infarction [4,7]. However, cases presenting with isolated pure alexia without accompanying aphasia, agraphia, or visual field defects are exceedingly rare. The present report contributes to this limited evidence by describing a case of pure alexia with intact writing and language, thereby emphasising the precision of lesion localisation and the role of functional disconnection in the pathophysiology.

This case highlights that uncommon cognitive syndromes can occasionally be the only clinical sign of an underlying cerebrovascular event. It emphasises the need for healthcare providers to maintain a high index of suspicion for stroke when encountering patients with isolated cognitive or visual-linguistic disturbances, especially those with known vascular risk factors. Prompt identification is crucial to initiate treatment swiftly and minimise the chances of future cerebrovascular incidents.

## Conclusions

This case underscores the importance of recognising isolated higher cortical dysfunctions - such as alexia without agraphia - as potential presentations of posterior circulation stroke. Despite the absence of motor or sensory deficits, the patient exhibited a profound and selective impairment in reading, reflecting a lesion in the left occipitotemporal region within PCA territory. Timely neuroimaging and clinical suspicion led to the correct diagnosis and initiation of appropriate acute stroke management.

Clinicians should remain vigilant when assessing patients with sudden-onset language or cognitive deficits, even in the absence of classic stroke signs. In patients with underlying vascular risk factors, such as atrial fibrillation or diabetes, an atypical symptom like acquired dyslexia should prompt urgent cerebrovascular evaluation. Pure alexia, though rare, serves as a powerful reminder of the brain’s functional compartmentalisation and the clinical value of thorough neurocognitive assessment in acute settings.
